# Chimeric Antigen Receptor T-Cell Therapy: A Beacon of Hope in the Fight Against Cancer

**DOI:** 10.7759/cureus.3486

**Published:** 2018-10-23

**Authors:** Syed Maaz Tariq, Syed Ali Haider, Mohammad Hasan, Amber Tahir, Maria Khan, Arisha Rehan, Anum Kamal

**Affiliations:** 1 Internal Medicine, Jinnah Sindh Medical University, Karachi, PAK; 2 Internal Medicine, Dow University of Health Sciences, Karachi, PAK

**Keywords:** car t-cell therapy, chimeric antigen receptor, immunomodulation, immune therapy, hematologic malignancies, solid tumors, axicabtagene ciloleucel, tisagenlecleucel

## Abstract

Despite significant advancements, relapses, and persistent malignancies are still a major challenge faced by the oncologists. Immunotherapy has shown remarkable potential in induction of sustained remission in refractory malignancies. Chimeric antigen receptor T-cell (CAR-T) therapy is a newer treatment methodology approved by the Food and Drug Administration (FDA). The chimeric pairing of an antigen receptor with the T-cell receptor (TCR) intracellular signaling domain allows cluster of designation 8 (CD8) cytotoxic T-cells to target cell surface makers independent of major histocompatibility complex (MHC) activation. Another essential feature which contributes to the broad applicability of CARs and expanding their potential targets is their ability to bind not only to proteins but also to carbohydrate and glycolipid structures. Their antigen-specific and targeted immune responses have shown promising outcomes in clinical trials particularly involving B-cell malignancies and solid tumors. High remission rates and low percentages of relapses have caused a paradigm shift in the treatment of relapsed or refractory cancers. Challenges include side effects such as cytokine release syndrome, on-target off-tumor toxicities, and replication of its success in treating solid tumors. The burden of side effects and hefty cost of treatment are major obstacles which could hinder its progress globally. Nevertheless, ongoing research would only result in a maximized therapeutic potential in addition to more patient- and cost-friendly treatment. In this review, we aim to provide the readers an overview of chimeric antigen receptor T-cell therapy, a relatively new advancement in the world of immuno-oncology and thereby also discussing its advantages, side effects and future challenges.

## Introduction and background

Conventionally, radiotherapy, and chemotherapy have been employed in the treatment of cancer for decades. Despite advancements regarding monoclonal antibodies and molecular therapeutics, relapses, toxicities, and unsustainable remissions have been few of the challenges that remain to be solved in cancer therapy. The cases of relapse and persistent malignancies are considered a setback in oncology which does not leave the clinicians with many options to consider.

Immunotherapy has proved itself as a beacon of light in the field of oncology. In the 1980s, Rosenberg and colleagues were first to show the potential of immunotherapy in treating malignancies by employing lymphokine-activated killer cells (LAK) produced by taking blood from the patients and treating their lymphocytes with interleukin 2 (IL-2) [1]. In this regard, the work of Gross and peers is also monumental. They exhibited that programming cytotoxic T cell to attack tumor cells is possible [2]. Their work arguably led to the foundation of the idea of chimeric antigen receptor (CAR) T-cell therapy. The rationale of using CAR-T cell therapy is its high affinity—100 times more than that of the native T-cell receptor (TCR). It results in the modification of T-cell lymphocytes such that they attack the cells which express this antigen.

## Review

Anti-tumor immunity

Anti-tumor immunity consists of two arms: innate immunity and adaptive immunity. Natural killer cells and myeloid cells, which make up the innate arm, recognize and destroy the virally infected cells and the tumor cells. The adaptive arm consists of B-cell and T-cell lymphocytes which are assisted by the antigen-presenting cells, for e.g., dendritic cells. Ehrlich, back in the 20th century, suggested that the immune system can prevent cancers. He envisioned antibodies as magic bullets that can attack malignancies without harming the organism in the process. His vision led to the production of monoclonal antibodies by Georges Kohler and Cesar Milstein. The success of these advancements was barred by problems in inducing immune effector mechanisms. This problem led to the development of chimeric and humanized antibodies using processes such as antibody-dependent, complement-dependent cytotoxicity, immunomodulation, and modulation of signal transduction [3].

T-cell engineering has opened the gates to new horizons in the discussion of anti-tumor immunity. It has helped in overcoming the drawback that T-cell response to tumors is ineffective as tumor cells express antigens which are also expressed by body tissues thereby preventing auto-immunity which, in Ehrlich’s words, is known as “horror autotoxicus” [4]. Genetic programming has made it possible to “enforce” tumor recognition, prolong survival, and expand T cell. Decades of hard work has come into fruition by the development of CARs, where each CAR T cell can kill numerous tumor cells by antigen release and promote the tumor lymphocytes to kill cancer cells. These engineered T cells are a new group of therapeutic agents which are ready to penetrate the saturated field of oncology as a ray of hope, especially in leukemias and lymphomas. Apart from killing cancer cells, there is a potential of them being useful in the field of infectious diseases and transplantation as well [5].

CAR T-cell therapy—how does it work?

CARs are hybrid receptors with three primary components:

A. Extracellular Domain

A ligand for a cell-surface molecule consisting of a single-chain variable fragment (scFv) derived from a monoclonal antibody or an antigen-binding fragment (Fab) joined by a flexible linker to signal domains assembled to redirect T-cell function [6]. It determines receptor selectivity and affinity and is similar to the light chain region of an antibody.

B. Transmembrane Domain

It connects the scFv region to costimulatory molecules and can influence the immunogenicity depending on its length.

C. Intracellular Domain

It is a tyrosine-based activation motif which transmits activation signals to T cells upon antigen binding. There is a hinge region which acts as a “spacer” between the extracellular domain and the transmembrane domain and helps in increasing conformational flexibility for antigen binding [7].

**Figure 1:**
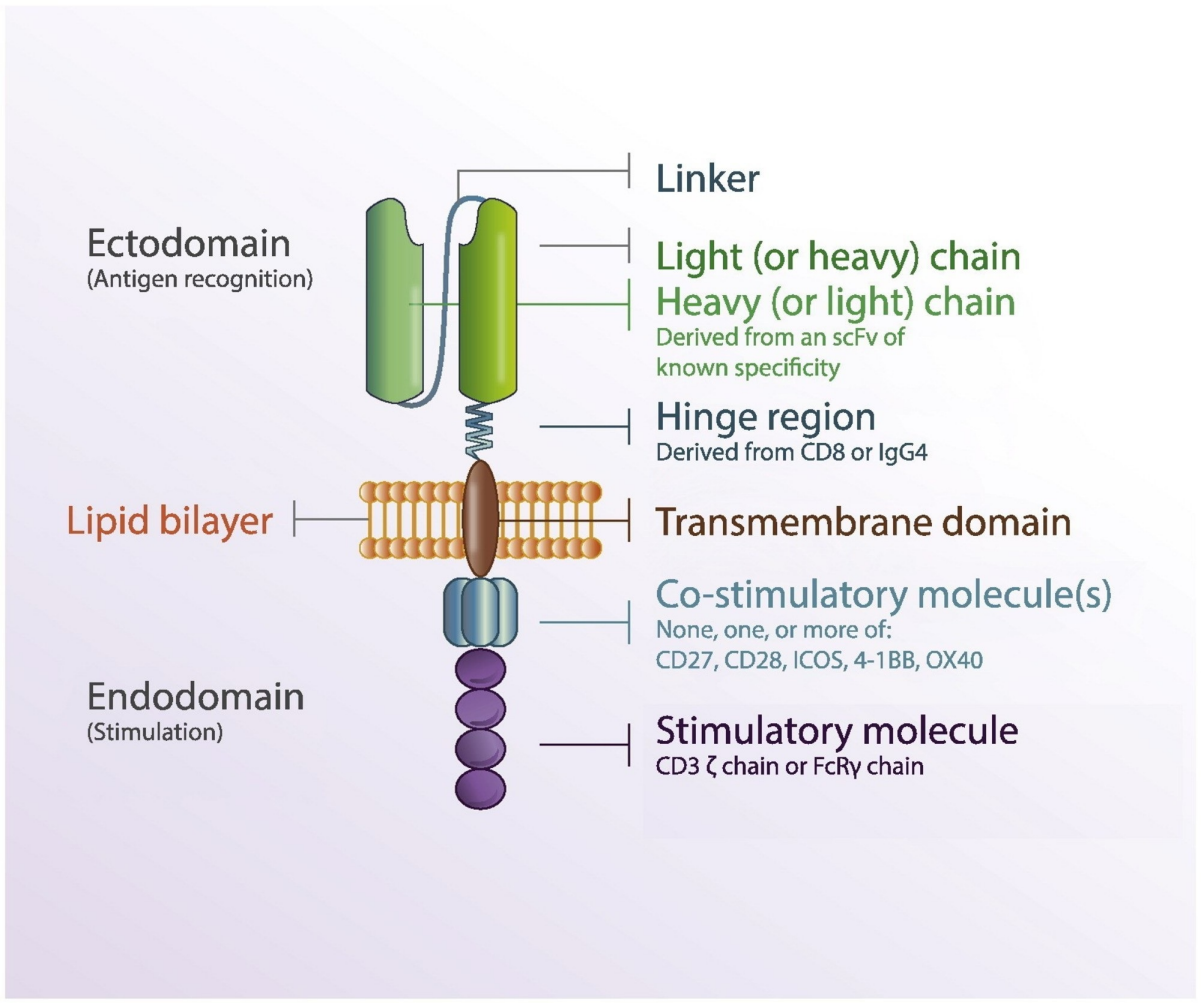
Anatomy of a chimeric antigen receptor. Reproduction permission obtained from the authors [8].

The structure of a CAR T Cell is shown in Figure 1.

The chimeric pairing of an antigen receptor with the T-cell receptor (TCR) and the consequent intracellular signaling allows CD8 cytotoxic T cells to target cell surface markers independent of major histocompatibility complex (MHC) activation [7]. Another essential feature which contributes to the broad applicability of CARs and expanding the potential targets is the ability of CAR T cells to bind not only to proteins but also to carbohydrate and glycolipid structures [8].

First-generation CARs include a single-chain variable region from a monoclonal antibody paired with an intracellular signaling domain, the CD3-ζ from the CD3 TCR or FcRγ and CD8, CD4, CD25, or CD16. These initiate phosphatidylinositol and tyrosine kinase pathways together with calcium influx in human T-cell leukemias. Although CD3-ζ chain aggregation is enough to conduct lytic activity in the cytotoxic T lymphocytes (CTL), the signal strength essential for cytotoxicity is still lower than that needed for other T-cell functions [7, 9]. This is probably why limited therapeutic responses have been reported with activating receptors and their anti-tumor effects are often limited to either local administration [10] or short-term systemic administration [11].

Second- and third-generation CAR T cells include co-activator domains (e.g., CD28, 4-1BB, OX-40) that further strengthen the T-cell activation signal, hence, boost the proliferation and production of the cytokines [7]. The first clinical study with third generation CAR T cells, however, did not show dramatic results [12]. Nonetheless, the potential strength of these “triple-decker” CARs needs more investigation to fully understand their signaling potential and promote sustained T cell function and survival, thereby preventing premature death, rapid exhaustion or undue proliferation.

**Figure 2:**
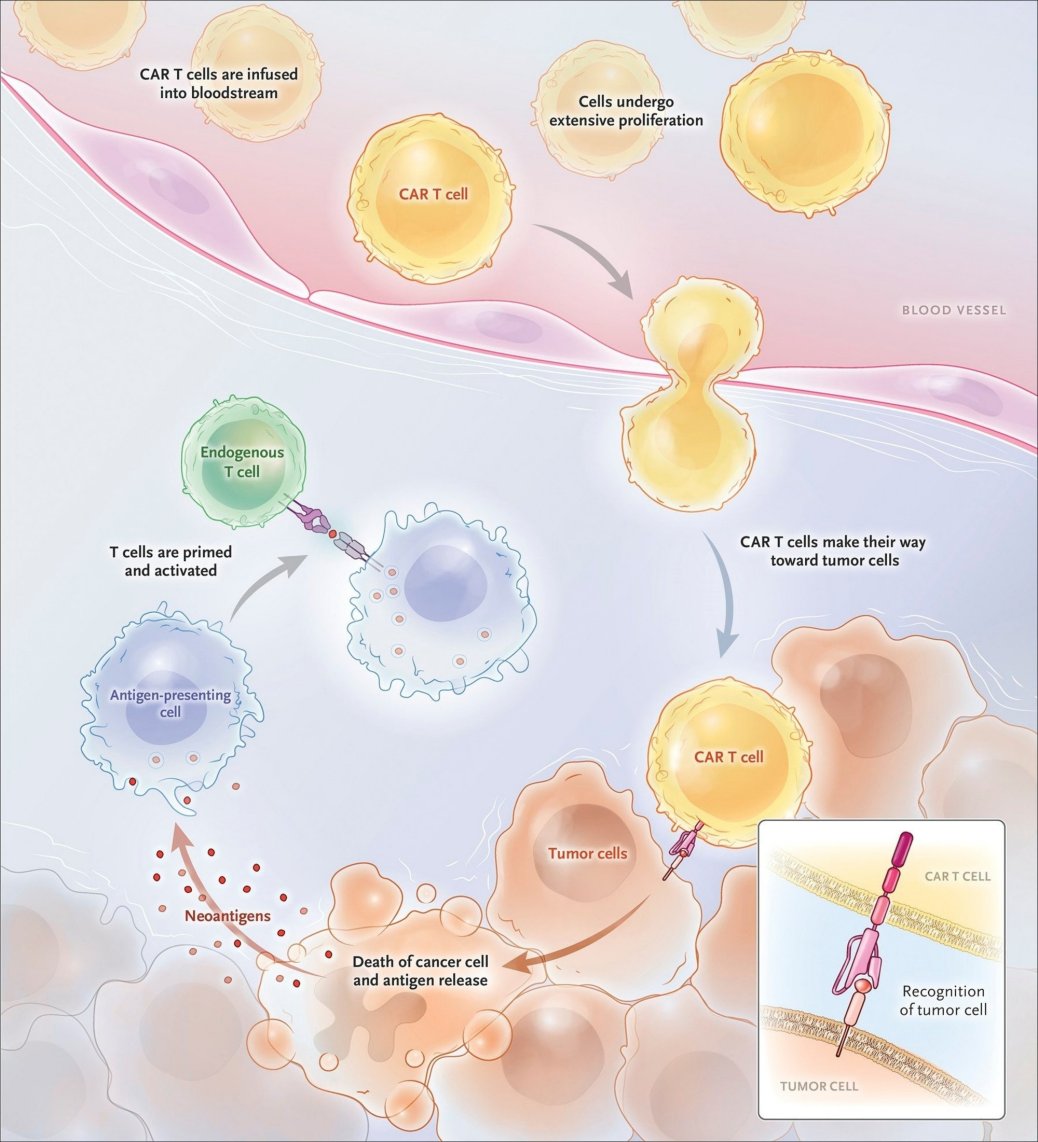
Chimeric antigen receptor (CAR) T cells engrafting, trafficking to tumor and proliferating extensively after infusion. Reproduction permission obtained from the authors [5].

To initiate CAR T-cell therapy, first T cells are collected from the patients’ blood in a procedure called leukapheresis. The T cells are then sent to the laboratory where they are subjected to genetic engineering which includes coupling of an anti-CD19 single-chain Fv domain to intracellular T-cell signaling domains of a T-cell receptor [13]. These re-engineered T cells are called “chimeric antigen receptor (CAR) T Cells.” They allow the T cells to recognize an antigen on targeted tumor cells. The re-engineered CAR T cells are then subjected to multiplication. When enough “expansion” has been achieved, these CAR T cells are frozen and then sent back to the center where the patient is being treated. This process takes almost two weeks. During these two weeks, the patient had been receiving a course of one or more chemotherapy agents known as “lymphodepletion”. The CAR T cells are thawed and infused into the patient. Once returned into the bloodstream, CAR T cells multiply in number. The entire process is shown in Figure 2.

These “attacker” cells then recognize, and attack, cells that have the targeted antigen on their surface. The CAR T cells may help guard against recurrence. They help in eradicating all of the cancer cells and have the ability to remain in the body for months guarding the patient against recurrence and long-term remissions [14].

CAR T-cell therapy for hematological malignancies

CAR T cell therapy has shown the most promising results in hematological malignancies. Although researchers had been experimenting with CAR T cell therapy for many years, it received its breakthrough with CD19 specificity for B cell malignancies. Groundbreaking researches were conducted since then and a number of clinical trials rose exponentially. There are two notable trials:

The ELIANA Trial

It was a multi-center, single-cohort, global study which showed a three-month overall remission rate (ORR) to be 81% with tisagenlecleucel in pediatric and young adult patients with CD19+ relapsed or refractory B-cell acute lymphoblastic leukemia (ALL). Survival probability was 73% at six months [15].

The ZUMA-1 Trial

It was a multicenter trial with diffuse large B-cell lymphoma (DLBCL), primary mediastinal B-cell lymphoma (PMBCL), or transformed follicular lymphoma (TFL) patients who had refractory disease despite undergoing recommended prior therapy. ORR achieved was 82%. The overall rate of survival at 18 months was 52% [16].

Other studies with ALL have shown complete remission rates from 70 to 90% [13, 17-18].

The combined results of these two major trials led the Food and Drug Administration (FDA) to approve two CAR T-cell therapy drugs [19]. These are:

A. Axicabtagene Ciloleucel

Approved for treatment of DLBCL, PMBCL, high-grade B-cell lymphoma, and TFL in adults.

B. Tisagenlecleucel

Approved for treatment of refractory and second or later relapsed cases of the B-cell precursor ALL in patients of up to 25 years. Adult relapsed or refractory cases of DLBCL, high-grade B-cell lymphoma and TFL after failure of two or more lines or therapy.

CAR T-cell therapy for solid tumors

The advent of CAR T-cell therapy in the treatment of hematological malignancies has changed the dimensions of cancer therapy to a great extent. Its application in the case of solid tumors remains to be a challenge. Although the FDA has not yet approved CAR T-cell therapy for solid tumors, the role of T cells in attacking solid tumors is established in the form of checkpoint therapy in infiltrating lymphocytes for malignancies like cholangiocarcinoma, colorectal carcinoma, and neuroblastoma [20-22]. Quite interestingly, CAR T-cell therapy was first tried to treat solid tumors, but the burden of “off-tumor” side effects has proved it to be a red flag in the conquest of further progress in this area. Presently, there has been an increasing number of clinical trials which have their efforts concentrated on targeting surface proteins of solid tumors like human epidermal growth factor receptor 2 (HER2), mesothelin, interleukin 13 receptor α (IL-13Rα), fibroblast activation protein (FAP), and carcinoembryonic antigen (CEA) [23]. Despite the hard work, the results have been demotivating so far. The factors responsible are not completely understood, but a few obstacles that are speculated to be the culprits are concisely described below:

Specificity of Target Antigen

First and foremost, the selection of the appropriate tumor-associated antigen (TAA) is arguably the most crucial component of CAR T-cell therapy. The TAA needs to be expressed on the surface of cancer cells but not on the normal tissue, otherwise such off-tumor side effects can occur that will prove to be endangering the patients’ life. The TAA should be expressed by each and every cancer cell.

The use of CD19 in CAR T cells constructed for B cell malignancies fulfills both of the criteria and virtually all of the leukemic cells express the antigen. There are a number of antigens which are being assessed for solid tumors but the results are discouraging so far [8]. It is worth mentioning that the phenomenon of immune-editing and tumor escaping is also a barrier. Cancers that occur due to viruses and the products expressed by these viruses can be an attractive target for CAR T cells, as these are not expressed by normal tissues [24].

Tumor Infiltration

After selection of the appropriate TAA, construction of CARs, and their infusion, the success of therapy hinges on proper tumor infiltration. Successful infiltration depends upon the interaction of adhesion receptors on T cell and tumor endothelium in addition to the match of chemokine receptor of CAR T cells (mainly CCR5, CXCR3) and chemokines secreted by the tumor.

Often the tumor produces insufficient quantities of CCR5, CXCR3 which results in ineffective targeting of tumor sites by CAR T cells. This obstacle can be dealt with by designing CAR T cells that co-express better-matched chemokine receptors [23].

Hostility of Tumor Micro-environment (TME)

After an appropriate choice of receptor and a successful invasion of the tumor, the microenvironment in the tumor itself poses a serious threat to the life of a CAR T cell. Many solid tumors are characterized by their stromal anatomic barriers. FAP can be used to reduce the number of tumor fibroblasts and help CAR T cells secrete an enzyme which can degrade matrix [25-26].

Although controversial, but the role of hypoxia in TME can be of importance. Evidence suggests that low oxygen can have a negative effect on T cell activity and can also decrease its proliferation. Hatfield has shown that high oxygen promotes anti-tumor immunity in mice and also reduces tumor growth [27]. Elevated lactate levels which promote acidosis and low glucose cause nutrient starvation inhibiting T cell proliferation [28-29]. Tumor cells and macrophages produce Prostaglandin E2 by the cyclooxygenase-2 enzyme. There are reports of Prostaglandin E2 inhibiting T cell proliferation [30].

Self-regulation of T cells

The expression of surface molecules like cytotoxic T-lymphocyte-associated protein 4 (CTLA-4) and Programmed Cell Death protein 1 (PD-1) have a negative effect on T cell activation. This has been reported in studies as well [31]. Fusion of an extracellular domain of PD-1 fused with cytoplasmic domain of the activated receptor is a promising solution to reverse PD-1 mediated inhibition of T cell [32]. Anti-CTLA-4 antibodies are also a promising solution to the CTLA-4 mediated inhibition of T cell activity [33]. Functioning of the T cell can also be hampered by activation-induced cell death. Efforts are being done to make the T cell express anti-apoptotic proteins such as BCL-2 [34].

Toxicities related to CAR T-cell therapy

With a great therapeutic potential comes a considerable burden of toxicities. The major safety concerns of this therapy are the self-destruction of normal tissue and an aggressive release of cytokines i.e. cytokine storm, which is linked with an accelerated immune response. These adverse effects are in contrast to those of cytotoxic lymphocytic antigen 4 (CTLA4) which are mainly rash and colitis etc. [35]. The spectrum of side effects depends upon the specificity of antibody scFv and activation of T cell. Various types of toxicities reported and studied in the literature are summarized below:

On-target Off-tumor

B cell aplasia was first predicted as on-target off-tumor adverse effect of CARs that target CD19, CD20, and CD22 [36]. This aplasia can be managed effectively by administering intravenous immunoglobulin. On-target off-tumor toxicity of CAR T cells was first reported in a clinical trial of renal cell carcinoma (Phase 1) patients treated with CAR T cells recognizing carbonic anhydrase IX (CAIX) on bile duct epithelium along with the malignancy resulting in cholestasis [37]. The most lethal on-target off-tumor toxicity reported in the literature is of a patient treated with ERBB2/HER2 specific third generation CAR T-cell for colorectal cancer. After initiation of the therapy, the patient went into respiratory distress, cardiac arrest, and died after five days. The expression of ERBB2, although in minute quantities, in lung epithelium became a reason of this fatal outcome [38].

Cytokine Release Syndrome (CRS)

Cytokine release syndrome is one of the most common side effects observed in CAR T-cell therapy [39]. The immune activation by the CAR T cells translates into the release of cytokines. Laboratory studies help in assessing the severity of CRS from mild (constitutional symptoms and/or grade-2 organ toxicity) to severe (grade ≥3 organ toxicity, aggressive clinical intervention, and/or potentially life-threatening). The clinical features include high fever, malaise, fatigue, myalgia, nausea, anorexia, tachycardia/hypotension, capillary leak, cardiac dysfunction, renal impairment, hepatic failure, and disseminated intravascular coagulation (DIC). Levels of cytokines including interferon-gamma, granulocyte macrophage colony-stimulating factor, IL-10, and IL-6 have been shown to rise following CAR T-cell infusion [40]. Currently, there are studies ongoing to assess the role of C-Reactive Protein, produced in response to IL-6 by the liver, as a severity marker for CRS [17]. Tocilizumab, an anti-IL-6 drug, approved by the FDA, is shown to immediately reverse the CRS effects [41]. In ELIANA trial, 77% of patients reported CRS, 48% of whom were managed with tocilizumab [15]. However, in ZUMA-1 trial, only 13% cases of CRS were seen [16].

Anaphylaxis

Humoral and cellular rejection of CAR T cells is well documented in the literature. This phenomenon could be attributed to the fact that antigen-binding domains of the modified T cells are derived from murine mAb [42]. Anaphylaxis due to CAR T-cell therapy was reported when, among four patients treated with mesothelin-specific CAR T cells, one developed cardiorespiratory failure which upon investigation revealed to be an IgE-mediated reaction [43]. Humanization of the CAR T cell components and proteins are a key to fight this lethal side effect.

Neurotoxicity

Neurotoxicity is well reported in the cases of B cell malignancies but fortunately, it is treatable [17]. The spectrum of neurotoxicity includes aphasia, seizure, obtundation, and myoclonus. Quite interestingly it is seen that the clinical activity shows seizure but the EEG readings suggest otherwise [40]. The possible mechanism behind this toxicity is yet to be understood as it is currently limited to CD19 CAR T cells only. Neurologic events were reported in 40% of patients in the ELIANA trial [15] and 28% in the ZUMA-1 trial [16].

## Conclusions

CAR T-cell therapy has immense therapeutic potential, especially in the domain of hematological malignancies where high remission rates have demonstrated feasibility and possibility of favorable outcomes in otherwise treatment-resistant cancers. Years of research and clinical trials have translated into significant clinical success. As far as the challenges are concerned, the foremost challenge faced by the oncologists is lack of a uniform and standard dose of transfusion cells. Researchers and oncologists have to work on obtaining the minimum dose to induce tumor antigen deletion and avoid CRS and tumor lysis syndrome. A challenge for clinical investigators is to treat CRS in such a way that the anti-tumor response of CAR T-cells is not affected. Use of corticosteroids is a potential treatment for CRS which does not dampen the anti-tumor properties of the infused T cells although prolonged use for more than 14 days may affect the anti-leukemic properties of CAR T cells. Reasonable targets must be set to maximally destruct the tumor but produce minimum toxicities. New targets and new drug combinations are needed to be experimented upon for hematological malignancies. If CAR-T therapy remains limited to only a few targets and blood cancers only, the efficacy of this therapy will be exhausted within a few years. Research is needed to identify better targets for solid tumors and to overcome obstacles in the tumor microenvironment that block T-cell functions, which would exponentially increase the efficacy and applicability of this therapy.
